# Supplementary material for: Versatile nitrate-respiring heterotrophs are previously concealed contributors to sulfur cycle
Source: Nat Commun. 2025 Jan 31;16:1202. doi: 10.1038/s41467-025-56588-1 (PMC11782648; doi:10.1038/s41467-025-56588-1)
Supplement: Supplementary file 1 — Supplementary Information [file 41467_2025_56588_MOESM1_ESM.pdf]

Supplementary Information for

Versatile nitrate-respiring heterotrophs are previously concealed contributors to sulfur cycle

Bo Shao[1], Yuan-Guo Xie[2], Long Zhang[3,4], Yang Ruan[5], Bin Liang[6], Ruochen Zhang[7], Xijun Xu[1], Wei Wang[1], Zhengda Lin[1], Xuanyuan Pei[8], Xueting Wang[1], Lei Zhao[1], Xu Zhou[6], Xiaohui Wu[9], Defeng Xing[1], Aijie Wang[6], Duu-Jong Lee[10], Nanqi Ren[1], Donald E. Canfield[11], Brian P. Hedlund[12,13], Zheng-Shuang Hua*[,2], Chuan Chen*[,1]

[1]State Key Laboratory of Urban Water Resource and Environment, School of Environment, Harbin Institute of Technology, Harbin, 150090, PR China
[2]Chinese Academy of Sciences Key Laboratory of Urban Pollutant Conversion, Department of Environmental Science and Engineering, University of Science and Technology of China, Hefei, 230026, PR China
[3]College of Life Sciences, Huaibei Normal University, 235000, Huaibei, PR China
[4]Department of Microbiology, Key Lab of Microbiology for Agricultural Environment, Ministry of Agriculture, College of Life Sciences, Nanjing Agricultural University, Nanjing 210095, PR China
[5]Jangsu Provincial Key Lab for Solid Organic Waste Utilization, Key Lab of Organic-based Fertilizers of China, Nanjing Agricultural University, Nanjing 210095, PR China
[6]State Key Laboratory of Urban Water Resource and Environment, School of Civil and Environmental Engineering, Harbin Institute of Technology Shenzhen, Shenzhen 518055, PR China
[7]School of Civil and Transportation, Hebei University of Technology, Tianjin 300401, PR China
[8]School of Environmental Engineering, Wuhan Textile University, Wuhan 430073, PR China
[9]School of Environmental Science and Engineering, Huazhong University of Science and Technology, Wuhan 430074, PR China
[10]Department of Mechanical Engineering, City University of Hong Kong, Tat Chee Avenue, Kowloon, Hong Kong, PR China
[11]Nordcee, Department of Biology, University of Southern Denmark, Odense, Denmark
[12]School of Life Sciences, University of Nevada, Las Vegas, Las Vegas, NV 89154, USA
[13]Nevada Institute of Personalized Medicine, Las Vegas, NV 89154, USA

***Corresponding authors**
Email: hzhengsh@ustc.edu.cn (Zheng-Shuang Hua); cchen@hit.edu.cn (Chuan Chen)

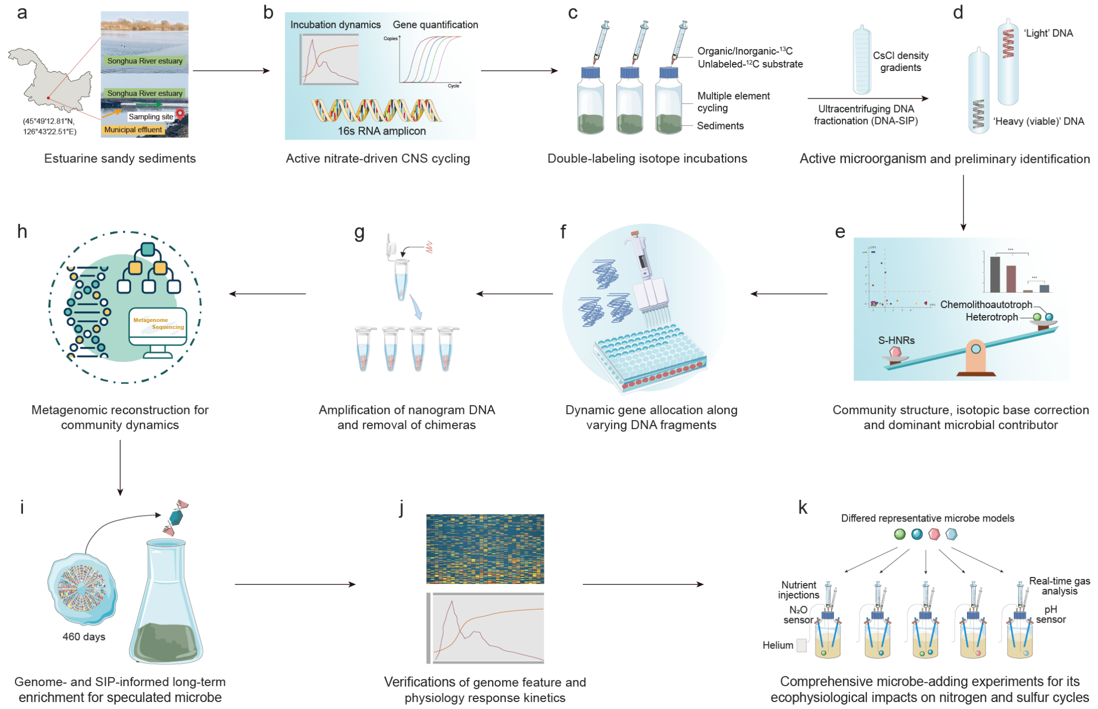

Supplementary Fig. 1 | Schematic of study components. a, Sampling site of estuarine sediments at the junction of effluent from municipal wastewater treatment plant (yellow) and the Songhua River (green). Arrows denote the flow direction of the water. The red coordinate symbol indicates the sampling location of this study. Sediment cores were sampled at 0–20 cm depth, with a water depth of 3.7 m **b,** Preliminary absolute gene quantification, amplicon sequencing and dynamic incubations for sediment activity tests. **c,** Short-term microcosm incubations with C, N, or S additions along with inorganic $^{13}C_i$- or organic $^{13}C_o$-carbon isotopes. **d,** Identification of active microorganisms by DNA stable isotope probing (DNA-SIP). **e,** Microbial function mapping and community contribution analysis of active microbial microorganisms after the community isotope response correction approach. **f,** Quantification of biomarkers involved in nitrogen and sulfur metabolism along DNA density gradients. **g,** Multiple displacement amplification and removal of chimeras in heavy DNA fractions. **h,** Metagenome-assembled genome reconstruction from heavy DNA fractions. **i,** Long-term enrichment of microorganisms informed based on genome- and SIP- results. **j,** Metagenome reconstruction and verification of reaction kinetics for the enrichments. **k,** Experimental incubations of the uncovered microorganisms for ecophysiological significance in biochemical cycling and greenhouse gas emissions.

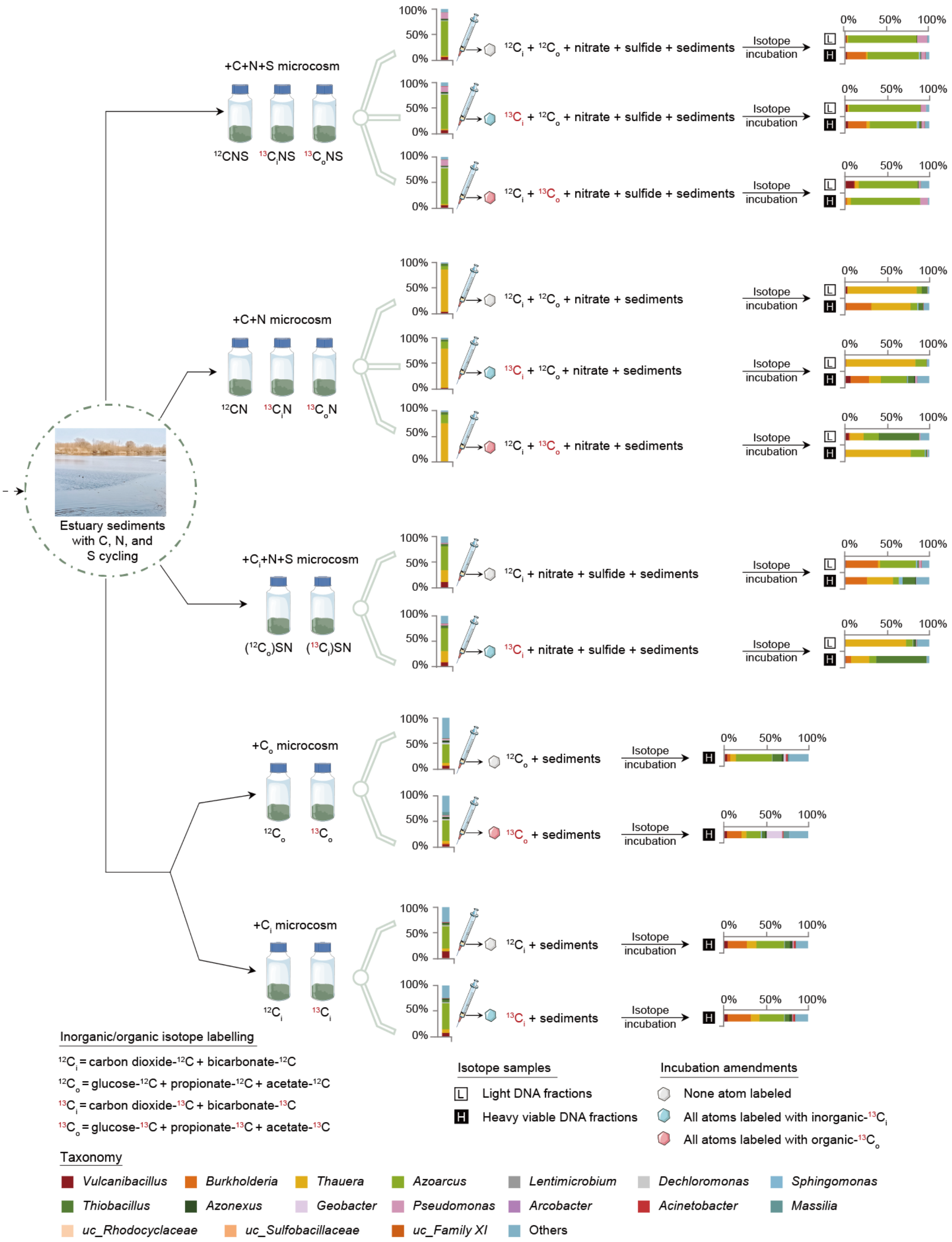

Supplementary Fig. 2 | Process workflow for isotope microcosm incubations. The sediment-containing bottles on the left represent the incubations designed to study ^{13}C assimilation within the context of coupled carbon, nitrogen, and sulfur cycles. Twelve bands near the isotope injector in the central panel represent the microbial community with short-term isotope incubation, while the right panel bands indicate the communities in heavy (H) and light (L) DNA fractions after isopycnic ultracentrifugation. For isotope injection, cyan and red hexagons represent the

inorganic $^{13}C_i$- and organic $^{13}C_o$-carbon substrates, while gray hexagons represent unlabelled substrates with both natural abundance $^{12}C_i$ and $^{12}C_o$.

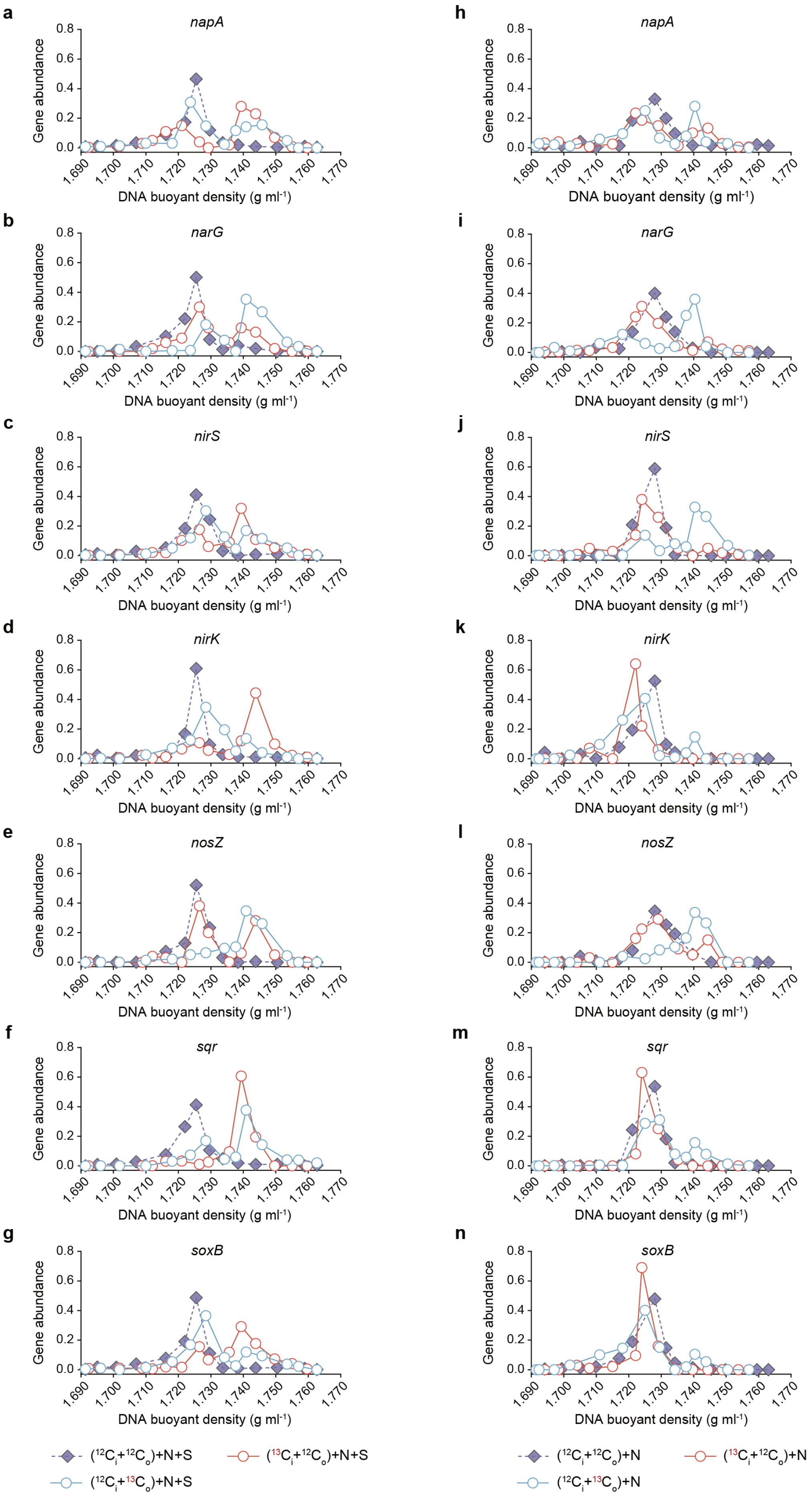
a
napA
b
narG
c
nirS
d
nirK
e
nosZ
f
sqr
g
soxB
h
napA
i
narG
j
nirS
k
nirK
l
nosZ
m
sqr
n
soxB
Gene abundance
0.0
0.2
0.4
0.6
0.8
1.690
1.700
1.710
1.720
1.730
1.740
1.750
1.760
1.770
DNA buoyant density (g ml-1)
(12Ci+12Co)+N+S
(13Ci+12Co)+N+S
(12Ci+13Co)+N+S
(12Ci+12Co)+N
(13Ci+12Co)+N
(12Ci+13Co)+N

Supplementary Fig. 3 | Complementary gradient gene quantification of key biomarkers in nitrogen and sulfur metabolisms. a-g, Dynamic abundance of functional genes along the DNA density gradient in CNS incubation microcosms. **h-n,** Dynamic abundance of functional genes along the DNA density gradient in CN incubation microcosms. For each buoyant density fraction, the gene abundance is normalized as a percentage of its copy number relative to the total DNA copy number (summed copies across all density fractions). All copies were amplified via absolute quantification. *napA*, cytochrome nitrate reductase; *narG*, nitrate reductase subunit alpha; *nirS*, cytochrome-cd_1 nitrite reductase; *nirK*, copper-containing nitrite reductase; *nosZ*, nitrous oxide reductase; *sqr*, sulfide:quinone oxidoreductase; and *soxB*, S-sulfosulfanyl-L-cysteine sulfohydrolase. Source data are provided as a Source Data file.

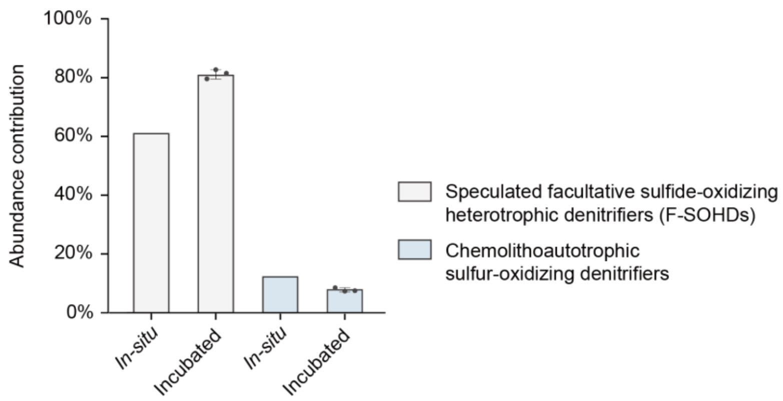

Supplementary Fig. 4 | Abundance contribution of chemolithoautotrophic sulfur-oxidizing denitrifiers and the inferred facultative sulfur-oxidizing heterotrophic denitrifiers (F-SOHDs) in sediment community. Community abundance was represented by the summed abundance of target genera inferred by community isotope response correction analysis on 16S rRNA amplicon. The community of natural *in-situ* sediments and short-term isotope incubation sediments were shown. Source data are provided as a Source Data file.

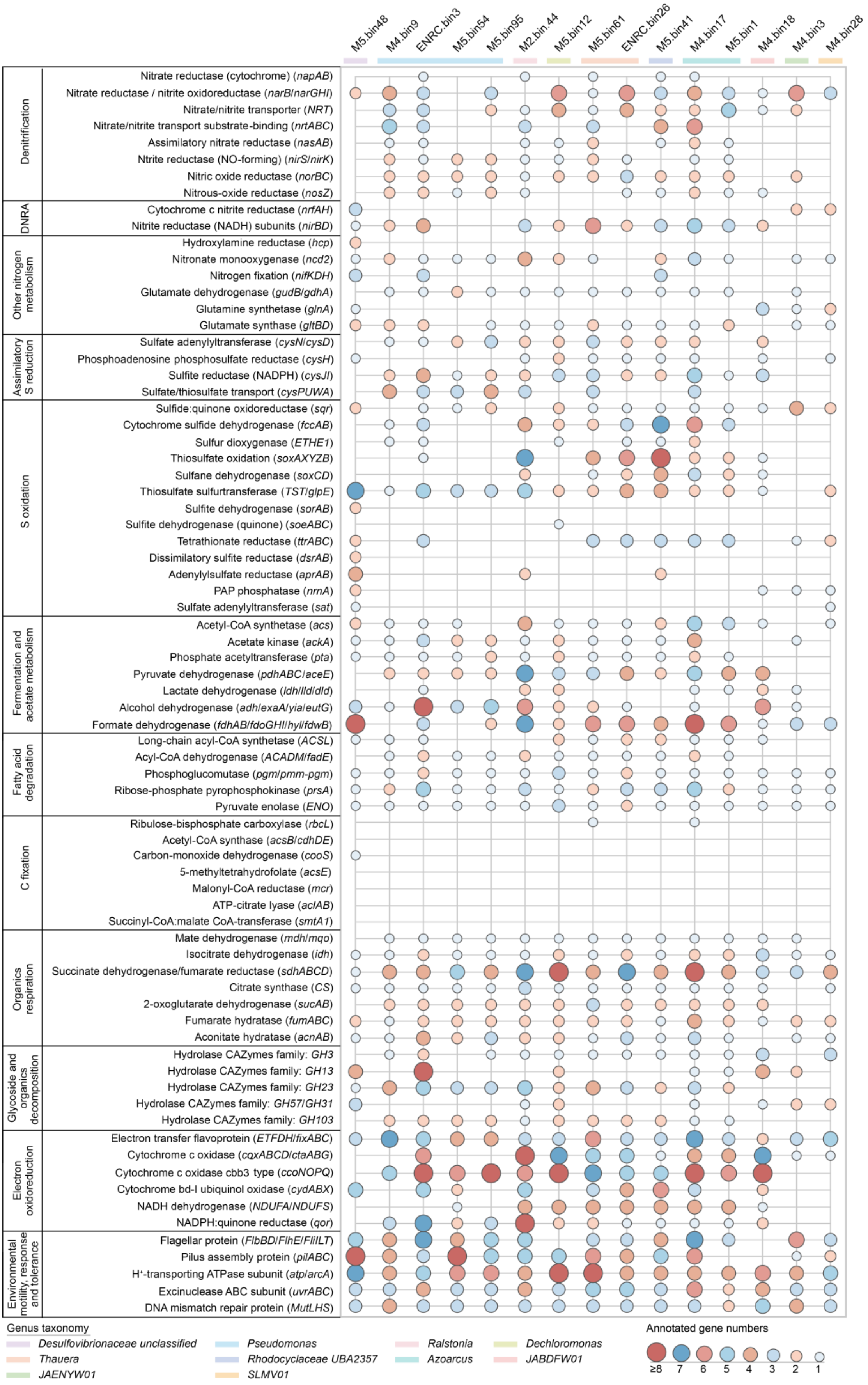

Supplementary Fig. 5 | Gene annotation of the representatives of identified

facultative sulfur-oxidizing nitrate-respiring heterotrophs from heavy $^{13}C_o$-assimilated DNA fractions. Top italics indicate the names of the representative high-quality genomes after reducing redundancy, while same color represents the MAGs that are taxonomically classified into a same taxon by Genome Taxonomy Database (GTDB)[1].The ENRC indicates the metagenome data of ENR_C4 enrichment. Predicted genes of carbon metabolism are divided into functional features of fermentation/acetate metabolism, fatty acid degradation, carbon fixation, organics respiration, glycoside/organics decomposition, and electron oxidoreduction. Additionally, the environmental adapting genes for motility, response and tolerance were shown. Source data are provided as a Source Data file.

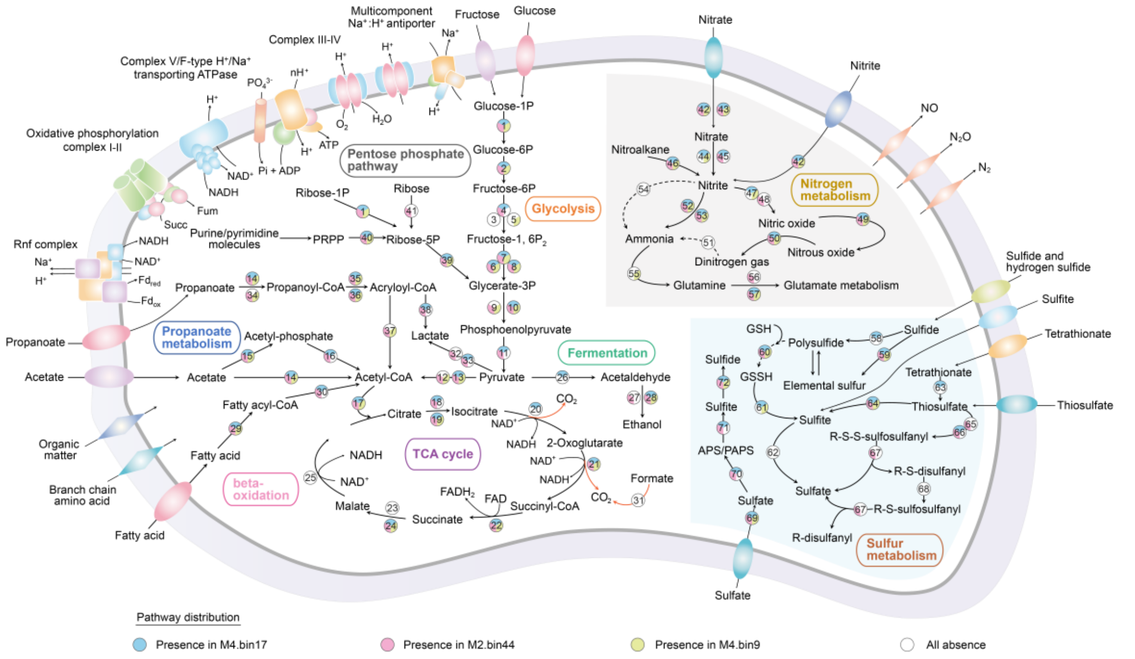

Supplementary Fig. 6 | Overview of metabolic potentials of versatile F-SOHD genomes in heavy $^{13}C_o$-assimilated DNA fractions. Metabolic pathways are shown based on genomic information from three MAGs of M4.bin.17 (*Azoarcus*), M2.bin44 (*Ralstonia*), and M4.bin9 (*Pseudomonas*). Detected pathways and genes related to organics fermentation, glycolysis, β-oxidation, acetate oxidation, propanoate metabolism, TCA cycle, nitrogen metabolism, sulfur metabolism, energy conservation and various electron/substrate transporters are shown. Solid/hollow area in trisected circle represents gene presence/absence in a target genome. Detailed full name and copy numbers of genes are recorded in Supplementary Table 3. The sixtieth pathway represents a non-enzyme-mediated spontaneous reaction[2]. GSSH glutathione persulfide, FAD flavin adenine dinucleotide, Fd ferredoxin, PRPP 5-Phospho-alpha-D-ribose 1-diphosphate. Source data are provided as a Source Data file.

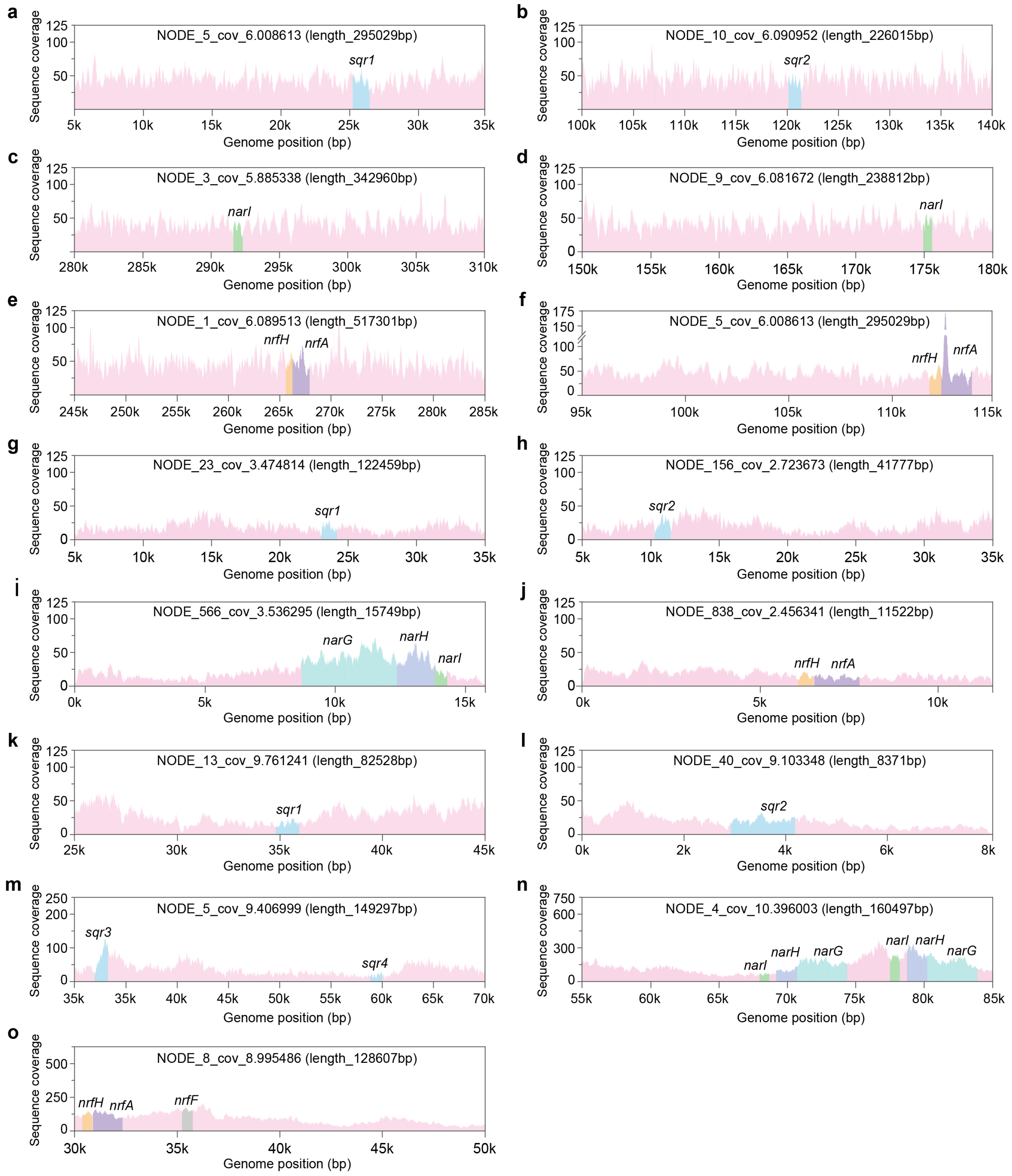

Supplementary Fig. 7 | Histograms information of the relatively even gene sequence coverage in three putative sulfur-oxidizing heterotrophic DNRA genomes. Scaffold length longer than 10000 bp is shown in each genome. In genomes, (**a**–**f**) for M5.bin48 (*uc_Desulfovibrionaceae*), (**g**–**j**) for M4.bin28 (*uc_SLMV01*), and (**k**–**o**) for M4.bin3 (*JAENYW01*) MAGs, respectively. Besides the sulfide-oxidizing *sqr* gene, two of big (*nrfA*) and small (*nrfH*) ammonium-producing cytochrome c-552 submits are indicated in scaffolds. The gene cluster of *narGHI* complex represents upstream nitrate reduction of DNRA process.

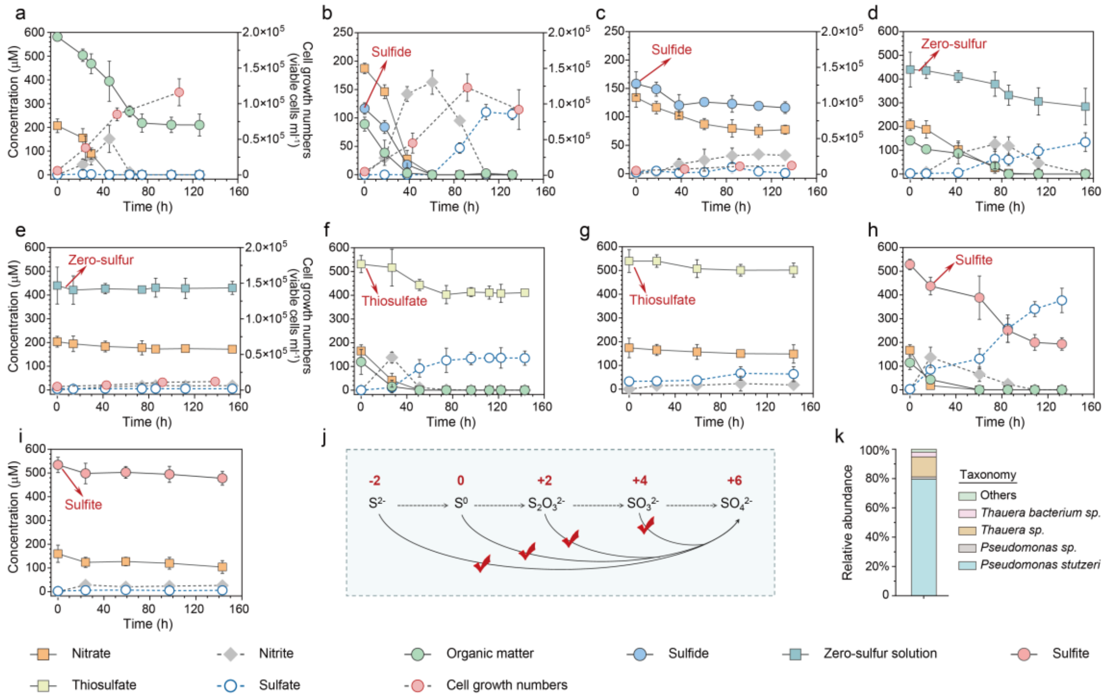

Supplementary Fig. 8 | Kinetic experiments, cell numbers, and microbial community in ENR_C4 enrichment. a-i, Reaction kinetics of sulfur oxidation and organic matter (OM) metabolisms accompanied by nitrate reduction. Different valence of sulfur compounds of reductive sulfide (-2), dissolved zero-valent sulfur (0), thiosulfate (+2), and sulfite (+4) were employed to test the speculated sulfur-supported heterotrophic denitrification metabolism. The zero-valent sulfur solution was prepared by the mixture of dissolved polysulfides and biological sulfur produced by *T. denitrificans* 25259. **j,** Completeness of sulfur-oxidizing metabolic pathways coupled to heterotrophic denitrification. Red tick indicates the presence of target sulfur oxidation capacities. **k,** Species-level community composition of ENR_C4 enrichment by full-length 16S rRNA gene amplicon library. Data from three replicates are presented as mean values +/- SEM.

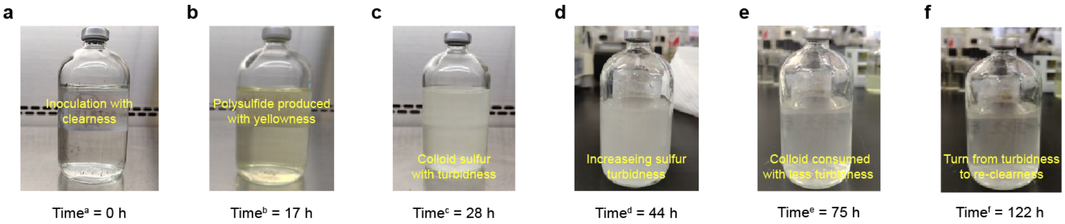

Supplementary Fig. 9 | Visualization of time-series liquid changes in ENR_C4 enrichment. a, Liquid state of the initial enrichment after inoculation into the medium. **b–d,** Liquid changes in the enrichment as sulfide was sequentially oxidized to polysulfides and white colloidal sulfur during heterotrophic denitrification. **e, f,** Liquid changes in the enrichment as produced colloidal sulfur was further oxidized to sulfate, accompanied by solution clearing over time. The yellow and white turbidness liquids denote the production of polysulfide and colloidal elementary sulfur, respectively, while other sulfur compounds of sulfide, sulfite, thiosulfate, and sulfate are colorless. All explanation of liquid color along intermediate production is supported by real-time physiochemical determinations.

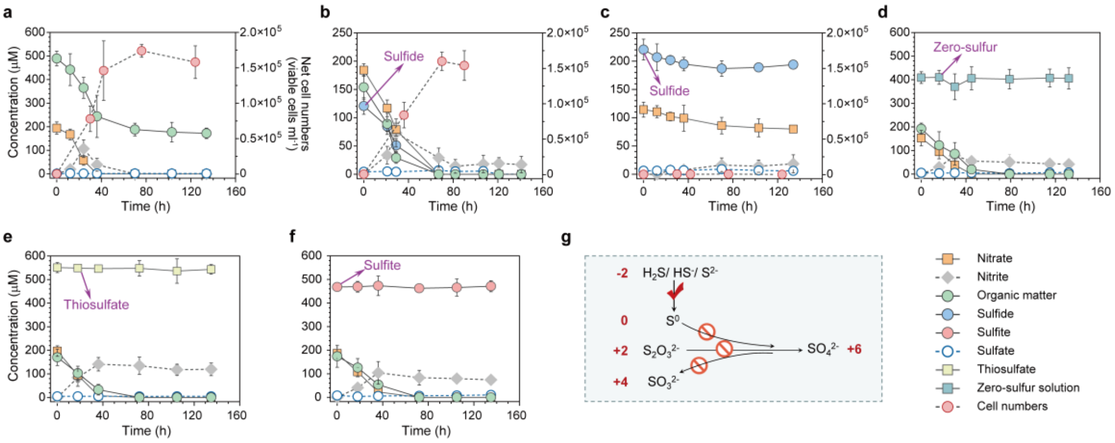

Supplementary Fig. 10 | Kinetic experiments and cell numbers in ENR_U2 enrichment. a-f, Reaction kinetics of sulfur oxidation and organic matter (OM) metabolisms accompanied by nitrate reduction. Different valence of sulfur compounds of reductive sulfide (-2), dissolved zero-valent sulfur (0), thiosulfate (+2), and sulfite (+4) were employed to test the speculated sulfur-supported heterotrophic denitrification metabolism. **g,** Completeness of sulfur-oxidizing metabolic pathways coupled to heterotrophic denitrification. Red tick/slash indicates the presence/absence of target sulfur oxidation capacities. Data from three replicates are presented as mean values +/- SEM.

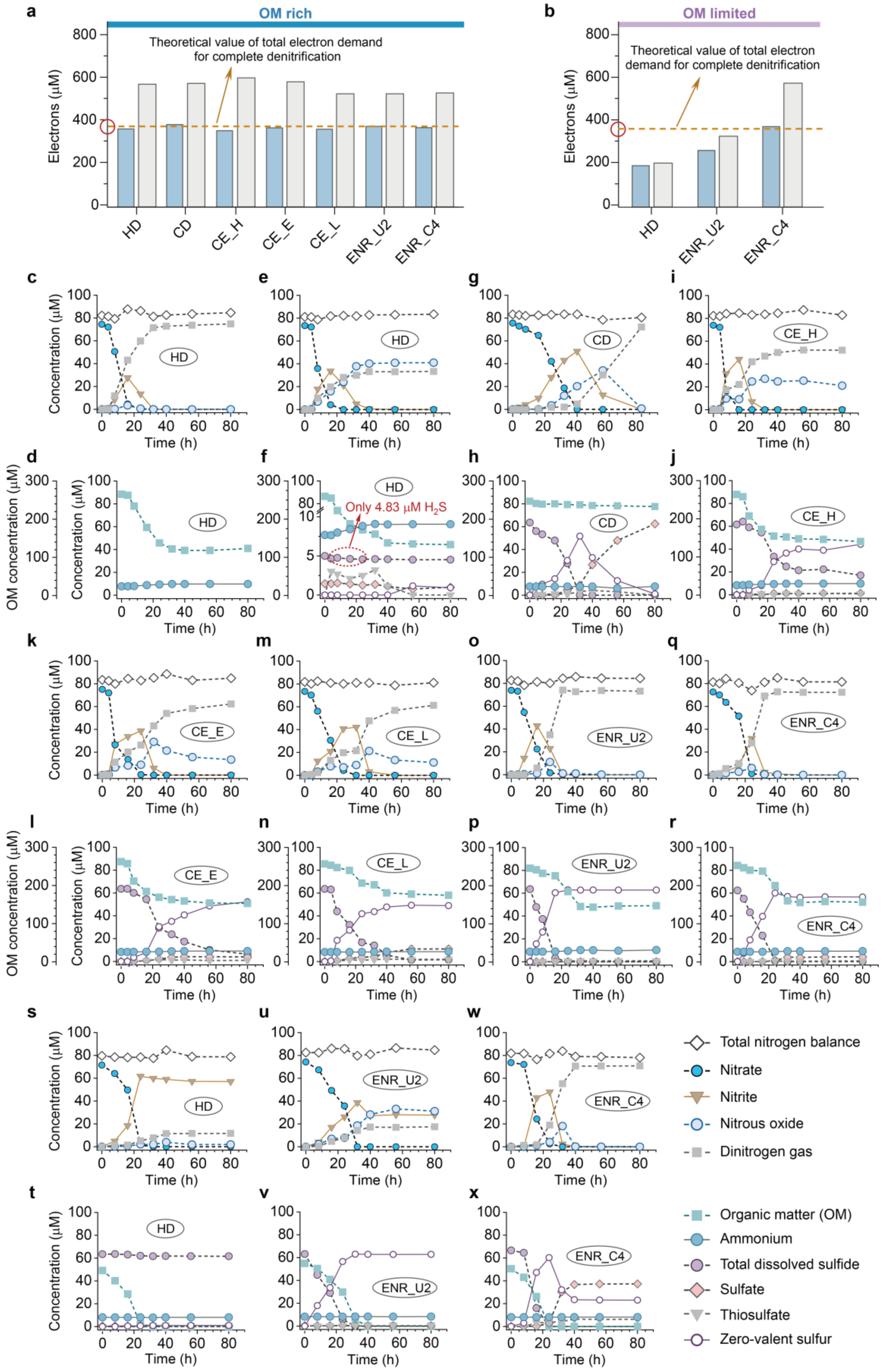

Supplementary Fig. 11 | Electron flow balance and comprehensive fate of element

biotransformation in ecophysiological incubation experiments. a, b, Electron budgets accounting for the electrons that were donated (gray boxes) and accepted (blue boxes) in different laboratory cultures under OM-rich and OM-limited conditions. Electron budgets were determined based on changes in concentrations of OM, nitrogen species (nitrate, nitrite, nitrous oxide, dinitrogen gas, and ammonium) and sulfur species (sulfide, zero-valent sulfur, thiosulfate, and sulfite). Dashed yellow lines represent the total theoretical electron demand for completely reducing nitrate to dinitrogen gas, assuming no electrons lost to anabolism. HD is a sediment enrichment culture containing conventional heterotrophic denitrifiers that cannot oxidize sulfide or other sulfur compounds. CD is a pure culture of the chemolithoautotrophic sulfur-oxidizing denitrifier *T. denitrificans* 25259, obtained from The Global Bioresource Center. The ENR_U2 and ENR_C4 are two enrichments dominated by F-SOHDs with capacity to oxidize sulfide only or capacity to oxidize sulfide, zero-valent sulfur, thiosulfate, and sulfite, respectively. To simulate the coexisted communities containing both non-sulfur-oxidizing conventional heterotrophic denitrifiers and sulfur-oxidizing chemolithoautotrophic denitrifiers, we established three different microbial communities by combining the HD culture with the CD *T. denitrificans* in ratios of 3:1 (CE_H, high ratio), 1:1 (CE_E, equal ratio), and 1:3 (CE_L, low ratio). **c**–**r,** Time-series biotransformation of nitrogen/sulfur species and OM consumption in OM-rich conditions. **s**–**x,** Time-series biotransformation of nitrogen/sulfur species and OM consumption in OM-limited conditions. For each group (such as **c, d**), top/below figures shows the data of nitrogen speciation and sulfur/OM, respectively.

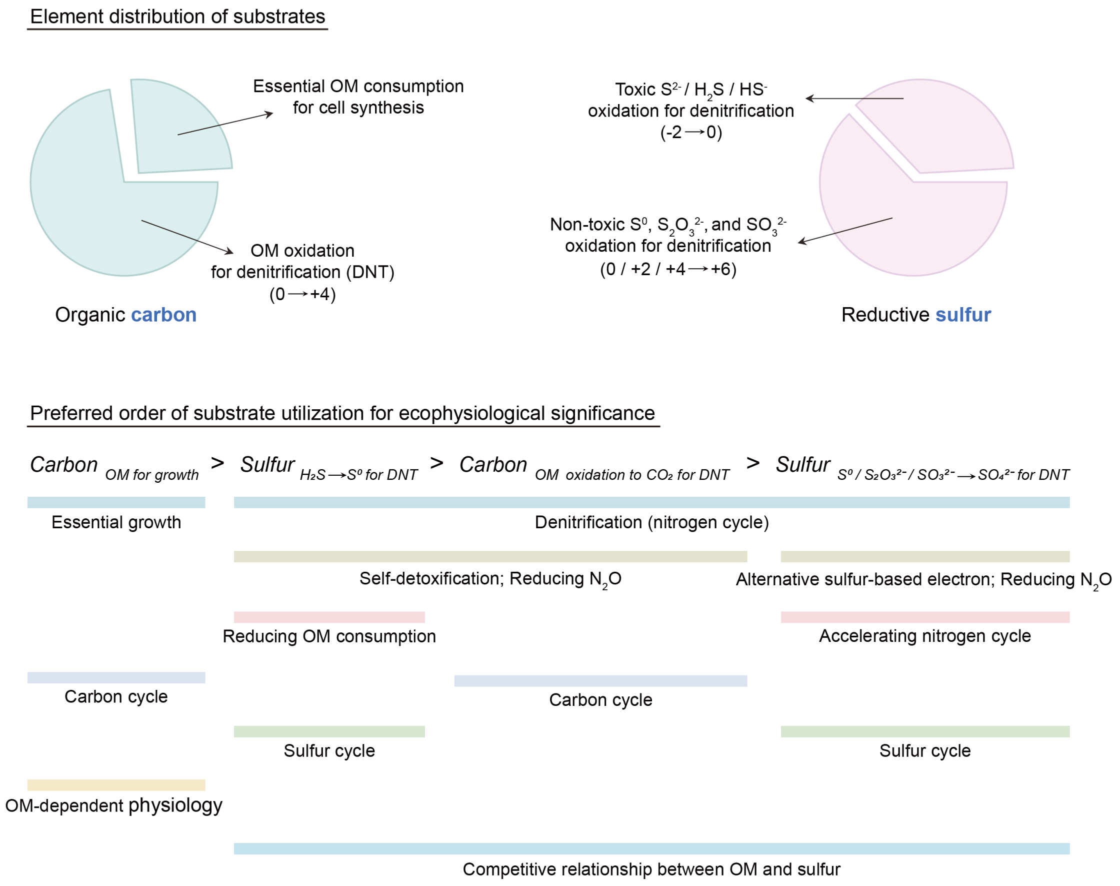

Supplementary Fig. 12 | Proposed schematic of element distribution and substrate utilization for F-SOHDs. Sector diagram represents the element distribution of two electron donors OM and sulfur within microbial reaction. The inequation below indicates the preferred order of substrate utilization, while the colored lines with varying length indicate the explanatory note for their ecophysiological significance. All results were derived from multiple evidences of isotope microcosm incubations, enrichment kinetics, ecophysiological incubations, and recalculated Gibbs free energy.

Supplementary Table 1. Summary statistics of the assembled moderate-/high-quality genomic bins from estuarine sediments, short-term inoculated sediments, isotope-assimilated DNA, and ENRC4 enrichment.

Bins	Completeness (%)	Contamination (%)	Strain heterogeneity (%)	Genome size (bp)	GC content (%)	No. of scaffolds	N50 value (bp)	No. of protein coding genes	Coding density (%)	No. of genes annotated by KO
M1.bin1	99.01	0	0	3324827	37.4	8	964866	2974	93.77	2243
M1.bin4	98.91	0	0	8195248	48.98	82	168286	6964	89.33	4675
M1.bin10	95.79	0.34	0	3984353	64.77	96	94436	3628	91.58	2842
M2.bin11	99.35	0.49	0	5046331	36.25	31	259477	4608	88.75	2970
M1.bin13	99.51	0.25	100	4371862	35.4	155	123358	4145	87.94	2607
M1.bin24	94.7	0.14	0	3762393	67	168	34482	3608	91.73	2737
M1.bin43	99.51	1.90	0	5574538	39.96	38	276887	4978	88.12	3349
M1.bin50	94.31	1.01	60	5836569	65.61	306	33875	5577	90.44	4545
M1.bin52	93.52	0.9	0	2376745	42.6	34	116314	2196	91.1	1379
M1.bin65	99.32	0.94	12.5	5615149	64.16	179	57103	5534	90.9	3822
M1.bin75	81.39	0.37	0	6187390	64.46	458	21268	6053	86.63	4589
M2.bin.7	97.59	1.78	0	4183317	49.04	155	55454	3730	89.48	2753
M2.bin20	91.55	1.76	25	4288538	61.28	172	54919	4093	90.57	3093
M2.bin44	97.21	0.92	0	5470547	63.55	60	155020	5127	89.45	4026
M2.bin48	82.21	0	0	4198168	59.16	204	47384	4192	89.41	3380
M2.bin50	98.13	0.10	100	5384542	62.96	89	90128	4873	88.02	3920
M2.bin53	94.51	0.90	33.33	4190033	41.67	239	30916	3773	87.32	2543
M2.bin54	93.38	0.89	57.14	2371439	60.09	87	55819	2286	90.11	1772
M2.bin84	93.93	0.53	33.33	2182143	43.31	60	51233	2151	89.94	1674

M4.bin3	85.32	1.54	0	3161232	34.47	60	130851	3157	87.67	2235
M4.bin9	92.8	0.95	0	3369950	60.79	248	26004	3361	90.55	2615
M4.bin17	97.1	0.27	50	4975972	62.74	372	24606	4989	90.78	3682
M4.bin18	98.95	0.25	0	5979119	43.72	116	102031	5063	87.1	3396
M4.bin28	94.06	0	0	2120920	41.43	83	52157	2210	89.46	1701
M5.bin1	81.64	0.18	100	2489730	62.98	221	17348	2486	91.13	2099
M5.bin4	86.83	0.15	100	1873506	59.85	405	4822	2024	92.08	1664
M5.bin7	98.34	0.68	0	3564224	65.98	83	91319	3404	92.53	2723
M5.bin11	82.57	0.21	0	2608274	60.23	170	24022	2495	93	1993
M5.bin12	98.74	0	0	4221609	60.62	184	37732	4263	89.21	3002
M5.bin15	85.36	0.47	0	2359178	65.82	168	23052	2403	90.37	1981
M5.bin16	98.58	1.74	66.67	2998214	65.57	140	31447	2968	89.77	2365
M5.bin20	95.7	0	0	3465269	42.69	61	96398	2757	90.34	2065
M5.bin25	97.07	0	0	4303779	57.02	378	17022	4308	89.99	3159
M5.bin29	92.08	0.21	100	2331314	55.12	294	9820	2523	92.04	1969
M5.bin31	91.5	2.62	73.33	3462445	64.11	631	7101	3662	87.95	2652
M5.bin34	100	0	0	2552625	46.73	14	347426	2430	90.79	1974
M5.bin39	100	0	0	4229738	58.85	28	223521	3811	90.33	3132
M5.bin41	99.09	0	0	3546985	63.93	112	48930	3176	91.48	2639
M5.bin46	98.67	0.12	100	1821269	34.47	120	27265	1777	91.51	1324
M5.bin47	98.71	0	0	3682465	62.58	57	108956	3457	91.27	2748
M5.bin48	99.41	0	0	4527070	65.33	34	260618	3907	88.59	2977
M5.bin52	98.37	2.57	15.38	2576830	26.72	143	31591	2700	94.02	2073
M5.bin54	92.65	2.77	77.78	3240104	48.57	290	16704	3199	88.73	2396
M5.bin61	96.44	0.06	100	3591736	66.41	278	22884	3459	92.2	2799

M5.bin63	84.76	0.58	0	2345277	56.56	46	96996	2283	91.71	1871
M5.bin64	98.77	1.23	20	3117759	63.58	46	132019	3066	91.45	2422
M5.bin70	99.31	0.95	0	3209487	63.32	95	72621	3293	89.74	2566
M5.bin71	81.41	0	0	2446791	57.34	148	25058	2327	88.19	1816
M5.bin72	86.07	0	0	3168774	37.34	454	8547	2813	86.62	1882
M5.bin81	97.24	0	0	3111241	57.45	42	173163	3001	93.37	2496
M5.bin83	92.06	1.38	0	3425164	64.8	95	56120	2896	90.73	2091
M5.bin84	98.67	0.89	0	1966718	34.25	23	129817	1837	93.02	1363
M5.bin89	98.6	0	0	2340361	35.12	63	61757	2319	91.6	1878
M5.bin92	99.19	0.29	100	4115350	39.38	243	39440	3471	91.1	2438
M5.bin94	98.89	0	0	3300831	66.22	184	26335	3345	91.51	2574
M5.bin95	96.74	4.93	62.5	3142686	48.79	287	21196	3091	88.07	2477
M5.bin97	100	0	0	1690168	36.99	49	67680	1704	93.32	1360
M5.bin98	97.31	0	0	3812419	42.21	34	217498	3135	89.31	2217
M5.bin103	93.02	0.46	50	3316710	68.1	212	35863	3146	92.72	2467
M5.bin104	85.18	0.91	0	3484728	40.5	234	21435	3351	90.64	2506
M5.bin112	87.28	0.65	0	2980694	30.67	441	8056	2877	89.04	1835
ENRC.bin3	96.52	0.49	80	6188144	63.62	421	29178	6146	89.58	4424
ENRC.bin26	96.83	0.21	100	3288783	65.98	187	28139	3168	91.89	2619

Supplementary Table 2. Primers and references of the eleven genes selected for qPCR quantification.

Target gene	Primer	Primer sequence (5'-3')	References
dsrA	DSR-1F+	ACSCACTGGAAGCACGCCGG	(Kondo et al. 2004)[3]
	DSR-R	GTGGMRCCGTGCAKRTTGG	
sqr	Forward	GCTCGGCAGCCTCAATAC	(Yin et al. 2014)[4]
	Reverse	GGTCGGACGGTGGTTACTG	
soxB	710F	ATCGGYCAGGCYTTYCCSTA	(Tourna et al. 2014)[5]
	1184R	MAVGTGCCGTTGAARTTGC	
nrfA	nrfAF2aw	CARTGYCAYGTBGARTA	(Welsh et al. 2014)[6]
	nrfAR1	TWNGGCATRTGRCARTC	
napA	V17m	TGGACVATGGGYTTYAAYC	(Bru et al. 2007)[7]
	napA-4r	ACYTCRCGHGCVGTRCCRCA	
narG	narG-f	TCGCCSATYCCGGCSATGTC	(Bru et al. 2007)[7]
	narG-r	GAGTTGTACCAGTCRGCSGAYTCSG	
nirS	nirS1F	TACCACCCSGARCCGCGCGT	(Braker et al. 1998)[8]
	nirS3R	GCCGCCGTCRTGVAGGAA	
nirK	876F	ATYGGCGGVCAYGGCGA	(Henry et al. 2004)[9]
	1040R	GCCTCGATCAGRTTRTGGTT	
norB	pF	CATGGCGCTGATAACGGG	(Dandie et al. 2007)[10]
	pR	CTTIACCATGCTGAAGGCG	
nosZ	Z2F	CGCRACGGCAASAAGGTSMSSGT	(Henry et al. 2006)[11]
	Z2R	CAKRTGCAKSGCRTGGCAGAA	
16S rRNA	341F	CCTACGGGNGGCWGCAG	(Herlemann et al. 2011)[12]
	805F	GACTACHVGGGTATCTAATCC	

Supplementary Table 3. Detailed full name and copy number of genes in metabolic pathways.

ID	KO	Gene or enzyme function	EC name	Sum copies
1	K15778	pmm-pgm; phosphomannomutase / phosphoglucomutase	5.4.2.8 5.4.2.2	3
2	K01810	GPI, pgi; glucose-6-phosphate isomerase	5.3.1.9	4
3	K03841	FBP, fbp; fructose-1,6-bisphosphatase I	3.1.3.11	0
4	K16370	pfkB; 6-phosphofructokinase 2	2.7.1.11	2
5	K21071	pfk, pfp; ATP-dependent phosphofructokinase / diphosphate-dependent phosphofructokinase	2.7.1.11 2.7.1.90	1
6	K01624	FBA, fbaA; fructose-bisphosphate aldolase, class II	4.1.2.13	5
7	K00134	GAPDH, gapA; glyceraldehyde 3-phosphate dehydrogenase	1.2.1.12	3
8	K00927	PGK, pgk; phosphoglycerate kinase	2.7.2.3	4
9	K15633	gpmI/gpmB; 2,3-bisphosphoglycerate-independent phosphoglycerate mutase	5.4.2.12	2
10	K01689	ENO, eno; enolase	4.2.1.11	3
11	K00873	PK, pyk; pyruvate kinase	2.7.1.40	3
12	K00906	aceE; pyruvate dehydrogenase E1 component	1.2.4.1	2
13	K00627	DLAT, aceF, pdhC; pyruvate dehydrogenase E2 component	2.3.1.12	7
14	K01895	ACSS1_2, acs; acetyl-CoA synthetase	6.2.1.1	10
15	K00925	ackA; acetate kinase	2.7.2.1	6
16	K00625	E2.3.1.8, pta; phosphate acetyltransferase	2.3.1.8	3
17	K01647	CS, gltA; citrate synthase	2.3.3.1	5
18	K01681	ACO, acnA; aconitate hydratase	4.2.1.3	3
19	K01682	acnB; aconitate hydratase 2 / 2-methylisocitrate dehydratase	4.2.1.3 4.2.1.99	3
20	K00031	IDH1, IDH2, icd; isocitrate dehydrogenase	1.1.1.42	1
21	K00164	OGDH, sucA; 2-oxoglutarate dehydrogenase E1 component	1.2.4.2	3

22	K01902	sucCD; succinyl-CoA synthetase subunit	6.2.1.5	3
23	K00239	sdhA, frdA; succinate dehydrogenase / fumarate reductase, flavoprotein subunit	1.3.5.1 1.3.5.4	0
24	K01676	E4.2.1.2A, fumA, fumB; fumarate hydratase, class I	4.2.1.2	3
25	K00024	mdh; malate dehydrogenase	1.1.1.37	0
26	K18366	bphJ, xylQ, nahO, tesF; acetaldehyde/propanal dehydrogenase	1.2.1.10 1.2.1.87	3
27	K13953	adhP; alcohol dehydrogenase, propanol-preferring	1.1.1.1	2
28	K00121	frmA, ADH5, adhC; S-glutathione dehydrogenase / alcohol dehydrogenase	1.1.1.284 1.1.1.1	3
29	K01897	ACSL, fadD; long-chain acyl-CoA synthetase	6.2.1.3	2
30	K00249	ACADM, acd; acyl-CoA dehydrogenase	1.3.8.7	2
31	K00122	FDH; formate dehydrogenase	1.17.1.9	0
32	K03778	ldhA; D-lactate dehydrogenase	1.1.1.28	1
33	K00782	lldEFG; L-lactate dehydrogenase complex protein LldG	N/A	3
34	K01908	ACSS3, prpE; propionyl-CoA synthetase	6.2.1.17	2
35	K00248	ACADS, bcd; butyryl-CoA dehydrogenase	1.3.8.1	4
36	K00252	GCDH, gcdH; glutaryl-CoA dehydrogenase	1.3.8.6	4
37	K01692	paaF, echA; enoyl-CoA hydratase	4.2.1.17	2
38	K01026	pct; propionate CoA-transferase	2.8.3.1	2
39	K00615	tktA, tktB; transketolase	2.2.1.1	6
40	K00948	PRPS, prsA; ribose-phosphate pyrophosphokinase	2.7.6.1	10
41	K00852	rbsK, RBKS; ribokinase	2.7.1.15	1
42	K02575	NRT, narK, nrtP, nasA; MFS transporter, NNP family, nitrate/nitrite transporter	N/A	6
43	K15576	nrtABCD, nasF, cynA; nitrate/nitrite transport system ATP-binding protein	N/A	4
44	K00370	narGHI, narZ, nxrA; nitrate reductase / nitrite oxidoreductase, alpha subunit	1.7.5.1 1.7.99.-	3
45	K02567	napA; nitrate reductase (cytochrome)	1.9.6.1	2
46	K00459	ncd2, npd; nitronate monooxygenase	1.13.12.16	9

47	K15864	nirS; nitrite reductase (NO-forming) / hydroxylamine reductase	1.7.2.1 1.7.99.1	3
48	K00368	nirK; nitrite reductase (NO-forming)	1.7.2.1	1
49	K04561	norB; nitric oxide reductase subunit B	1.7.2.5	3
50	K00376	nosZ; nitrous-oxide reductase	1.7.2.4	5
51	K02586	nifDKH; nitrogenase molybdenum-iron protein alpha chain	1.18.6.1	0
52	K00362	nirB; nitrite reductase (NADH) large subunit	1.7.1.15	6
53	K00363	nirD; nitrite reductase (NADH) small subunit	1.7.1.15	4
54	K03385	nrfAH; nitrite reductase (cytochrome c-552)	1.7.2.2	0
55	K01915	glnA, GLUL; glutamine synthetase	6.3.1.2	0
56	K00260	GDH2; glutamate dehydrogenase	1.4.1.2	1
57	K15371	gltBD; glutamate synthase (NADPH)	1.4.1.13	3
58	K17218	sqr; sulfide:quinone oxidoreductase	1.8.5.4	1
59	K17230	fccA; cytochrome subunit of sulfide dehydrogenase	N/A	9
60	-	Unindentified spontaneous reaction processing glutathione to glutathione persulfide intermediate	-	-
61	K17725	ETHE1; sulfur dioxygenase	1.13.11.18	3
62	K05301	sor; sulfite dehydrogenase	1.8.2.1	0
63	K08357	ttrABC; tetrathionate reductase	N/A	1
64	K01011	TST, MPST, sseA; thiosulfate/3-mercaptopyruvate sulfurtransferase	2.8.1.1 2.8.1.2	5
65	K17222	soxA; L-cysteine S-thiosulfotransferase	2.8.5.2	1
66	K17226	soxY; sulfur-oxidizing protein SoxY	N/A	3
67	K17224	soxB; S-sulfosulfanyl-L-cysteine sulfohydrolase	3.1.6.20	1
68	K17225	soxC; sulfane dehydrogenase subunit SoxC	1.8.2.6	0
69	K02046	cysU; sulfate/thiosulfate transport system permease protein	N/A	3
70	K00956	cysN; sulfate adenylyltransferase subunit 1	2.7.7.4	2

71	K00390	cysH; phosphoadenosine phosphosulfate reductase	1.8.4.8 1.8.4.10	2
72	K00381	cysI; sulfite reductase (NADPH) hemoprotein	1.8.1.2	3

Supplementary References

1 Parks, D. H. et al. A complete domain-to-species taxonomy for Bacteria and Archaea. *Nat. Biotechnol.* **38**, 1079-1086 (2020).

2 Xia, Y. et al. Sulfide production and oxidation by heterotrophic bacteria under aerobic conditions. *ISME J.* **11**, 2754-2766 (2017).

3 Kondo, R., Nedwell, D. B., Purdy, K. J. & Silva, S. Q. Detection and Enumeration of Sulphate-Reducing Bacteria in Estuarine Sediments by Competitive PCR. *Geomicrobiol J.* **21**, 145-157 (2004).

4 Yin, H. et al. Whole-genome sequencing reveals novel insights into sulfur oxidation in the extremophile *Acidithiobacillus thiooxidans*. *BMC Microbiol.* **14**, 179 (2014).

5 Tourna, M., Maclean, P., Condron, L., Callaghan, M. & Wakelin, S. A. Links between sulphur oxidation and sulphur-oxidising bacteria abundance and diversity in soil microcosms based on *soxB* functional gene analysis. *FEMS Microbiol. Ecol.* **88**, 538-549 (2014).

6 Welsh, A., Chee-Sanford, J. C., Connor, L. M., Löffler, F. E. & Sanford, R. A. Refined *NrfA* Phylogeny Improves PCR-Based *nrfA* Gene Detection. *Appl. Environ. Microbiol.* **80**, 2110-2119 (2014).

7 Bru, D., Sarr, A. & Philippot, L. Relative abundances of proteobacterial membrane-bound and periplasmic nitrate reductases in selected environments. *Appl. Environ. Microbiol.* **73**, 5971-5974 (2007).

8 Braker, G. & Kp, F. A. W. Development of PCR primer systems for amplification of nitrite reductasegenes (*nirK* and *nirS*) to detect denitrifying bacteria in environmentalsamples. *Appl. Environ. Microbiol.* **64**, 3769-3775 (1998).

9 Henry, S. et al. Quantification of denitrifying bacteria in soils by *nirK* gene targeted real-time PCR. *J. Microbiol. Methods.* **59**, 327-335 (2004).

10 Dandie, C. E. et al. Nitric oxide reductase-targeted real-time PCR quantification of denitrifier populations in soil. *Appl. Environ. Microbiol.* **73**, 4250-4258 (2007).

11 Henry, S., Bru, D., Stres, B., Hallet, S. & Philippot, L. Quantitative detection of the *nosZ* gene, encoding nitrous oxide reductase, and comparison of the abundances of 16S rRNA, *narG*, *nirK*, and *nosZ* genes in soils. *Appl. Environ. Microbiol.* **72**, 5181-5189 (2006).

12 Herlemann, D. P. R. et al. Transitions in bacterial communities along the 2000 km salinity gradient of the Baltic Sea. *ISME J.* **5**, 1571-1579 (2011).
