# Supplementary material for: Versatile nitrate-respiring heterotrophs are previously concealed contributors to sulfur cycle
Source: Nat Commun. 2025 Jan 31;16:1202. doi: 10.1038/s41467-025-56588-1 (PMC11782648; doi:10.1038/s41467-025-56588-1)
Supplement: Supplementary file 2 — Reporting Summary [file 41467_2025_56588_MOESM2_ESM.pdf]

nature portfolio

Corresponding author(s): Chuan Chen, Zheng-Shuang Hua

Last updated by author(s): Jan 9, 2025

Reporting Summary

Nature Portfolio wishes to improve the reproducibility of the work that we publish. This form provides structure for consistency and transparency in reporting. For further information on Nature Portfolio policies, see our Editorial Policies and the Editorial Policy Checklist.

Statistics

For all statistical analyses, confirm that the following items are present in the figure legend, table legend, main text, or Methods section.

n/a	Confirmed	
☐	☒	The exact sample size (*n*) for each experimental group/condition, given as a discrete number and unit of measurement
☐	☒	A statement on whether measurements were taken from distinct samples or whether the same sample was measured repeatedly
☐	☒	The statistical test(s) used AND whether they are one- or two-sided<br>*Only common tests should be described solely by name; describe more complex techniques in the Methods section.*
☒	☐	A description of all covariates tested
☐	☒	A description of any assumptions or corrections, such as tests of normality and adjustment for multiple comparisons
☐	☒	A full description of the statistical parameters including central tendency (e.g. means) or other basic estimates (e.g. regression coefficient) AND variation (e.g. standard deviation) or associated estimates of uncertainty (e.g. confidence intervals)
☐	☒	For null hypothesis testing, the test statistic (e.g. *F*, *t*, *r*) with confidence intervals, effect sizes, degrees of freedom and *P* value noted<br>*Give P values as exact values whenever suitable.*
☐	☒	For Bayesian analysis, information on the choice of priors and Markov chain Monte Carlo settings
☐	☒	For hierarchical and complex designs, identification of the appropriate level for tests and full reporting of outcomes
☒	☐	Estimates of effect sizes (e.g. Cohen's *d*, Pearson's *r*), indicating how they were calculated

Our web collection on statistics for biologists contains articles on many of the points above.

Software and code

Policy information about availability of computer code

Data collection

No software was used for data collection.

Data analysis

Open source tools:
1. Sequences cluster: Mothur (v1.34.3)
2. Taxonomic sequence assignment: SILVA (v132)
3. Metagenomic sequence assembly: SPAdes (v.3.15)
4. Genome binning: MaxBin2 (v.2.2.7), CONCOCT (v.1.1.0), MetaBAT (v.2.12.1), DASTool (v.1.1.1)
5. Genome bins quality evaluation: CheckM (v.1.1.3)
6. Genome dereplication: dRep (v.3.2.2)
7. Taxonomic assignment of genomes: GTDB-Tk (v.2.0.0)
8. Poorly alignment regions remover: TrimAl (v.1.4.rev22)
9. Phylogenetic tree construction: IQ-TREE (v.1.6.12)
10. Gene prediction: Prodigal (v.2.6.3)
11. Marker gene recruitment: AMPHORA (v.2.0)
12. Gene search against: KofamScan (v.1.3.0)
13. Functional annotation: DIAMOND (v.2.0.11.149)
14. Sequence alignment: MUSCLE (v.3.8.31)
15. Tree visualization: iTOL (v.6)
16. Statistical analyses: R platform (http://cran.r-project.org)

For manuscripts utilizing custom algorithms or software that are central to the research but not yet described in published literature, software must be made available to editors and reviewers. We strongly encourage code deposition in a community repository (e.g. GitHub). See the Nature Portfolio guidelines for submitting code & software for further information.

Data

Policy information about availability of data

All manuscripts must include a data availability statement. This statement should provide the following information, where applicable:

- Accession codes, unique identifiers, or web links for publicly available datasets
- A description of any restrictions on data availability
- For clinical datasets or third party data, please ensure that the statement adheres to our policy

The sequence data of 16S rRNA amplicons, metagenomes, and metagenome-assembled genomes generated in this study have been deposited in the NCBI Sequence Read Archive under Bioproject PRJNA1108931 (https://www.ncbi.nlm.nih.gov/sra/?term=PRJNA1108931). The detailed information of genomes is provided in Supplementary Table 1. Relevant data are available within the paper and source data files. Source data are provided with this paper.

Research involving human participants, their data, or biological material

Policy information about studies with human participants or human data. See also policy information about sex, gender (identity/presentation), and sexual orientation and race, ethnicity and racism.

Reporting on sex and gender	N/A
Reporting on race, ethnicity, or other socially relevant groupings	N/A
Population characteristics	N/A
Recruitment	N/A
Ethics oversight	N/A

Note that full information on the approval of the study protocol must also be provided in the manuscript.

Field-specific reporting

Please select the one below that is the best fit for your research. If you are not sure, read the appropriate sections before making your selection.

☐ Life sciences ☐ Behavioural & social sciences ☒ Ecological, evolutionary & environmental sciences

For a reference copy of the document with all sections, see nature.com/documents/nr-reporting-summary-flat.pdf

Ecological, evolutionary & environmental sciences study design

All studies must disclose on these points even when the disclosure is negative.

Study description	The main point of this study is to reveal the microbial coupling between nitrogen and sulfur cycling and its contribution on greenhouse gas emissions in aquatic sediments. This study involved several types of data: 1) amplicons, metagenomes and metagenome-assembled genomes; 2) DNA-stable isotope probing using different inorganic 13Ci and organic 13Co isotopes; 3) quantitative PCR for genes involved in denitrification and sulfide oxidation. The physicochemical data for all sediments were obtained through three biological replicates.
Research sample	Our study samples were collected from the organic-rich estuarine sediments of the Songhua River in Heilongjiang Province, China. The samples were then used for long-term enrichment of specific microorganisms in the laboratory with custom-prepared media containing nitrate, organic carbon, sulfide, sulfate, and trace elements.
Sampling strategy	Approximately 200 mL of sediment samples were collected using a gravity core sampler and stored in 500 mL pre-sterilized sealed glass bottles. Helium was used to flush the bottles to maintain an anaerobic environments before transporting to the lab. For all experiments of microbial incubation, the sediments were randomly inoculated into anaerobic bottles after being mixed.
Data collection	One overlying water sample was fixed immediately using sulfide antioxidant buffer to prevent sulfide oxidation, while in another sample sulfide was precipitated by zinc chloride to filter for other chemical analyses. The concentrations of sulfate, nitrate, and nitrite were measured using an ICS-3000 ion chromatograph (Dionex, USA). Ammonium was determined by the standard Nessler reagent method. Total organic carbon was determined using a multi N/C 3100 (Analytik Jena, Germany). DNA was extracted from 0.25 g of sediment using PowerSoil DNA isolation kit (MoBio, USA). Ten genes involved in sulfate reduction (dsrA), sulfide oxidation (sqr, soxB), nitrate reduction (napA, narG), denitrification (nirS, nirK, norB, nosZ), and DNRA (nrfA) were quantified. DNA concentrations were determined using the Quant-iT PicoGreen dsDNA kit (Thermo Scientific, USA). Recombinant plasmids with a cloned target gene were tenfold diluted for an eight-point standard calibration curves ranging from10 to108. The raw data were recorded by B.S. and Z.D.L using pen and paper for subsequent analysis.

Timing and spatial scale	Five replicate sediments were collected in August of 2019. The detailed locations of the samples have been shown in Supplementary Fig. 1 in the Supporting Information.
Data exclusions	No data was excluded.
Reproducibility	All sample collection procedures and experimental conditions were described in Methods as much detail as possible to enhance reproducibility. Our experiment aimed to elucidate the microbial mechanisms involved in denitrification-driven carbon, nitrogen, and sulfur cycling. However, the addition of relatively high concentrations of isotope-labeled substrates in the DNA-stable isotope probing process may introduce subtle shifts, making it difficult to replicate the community composition completely. Additionally, the mutiple environmental factors such as variations in the composition and abundance of functional microorganisms within the community, sediment sand content, and incubation temperature during the microcosm process can make it challenging to fully replicate the experiment. Despite this, the overall trends observed in the repeated experiments were consistent.
Randomization	For all experiments of microbial incubation, the collected sediment samples were thoroughly mixed by shaking and then randomly inoculated into sediment incubations and microcosm experiments. In all experiments, sediments and water samples were randomly collected for DNA extraction and subsequent physicochemical analysis.
Blinding	Mis-assembled scaffolds introduced by assemblers may result in the blinding. Also, bias might be existed during the genome binning step. To avoid this, we used several binning tools and adopted DasTool to select the best bin.

Did the study involve field work? ☒ Yes ☐ No

Field work, collection and transport

Field conditions	The temperature of the sediment habitat ranged from 19.2 to 24.8°C, with pH values between 7.03 and 7.16
Location	Samples were collected from the organic-rich estuarine sediments of the Songhua River (45°49′12.81″N, 126°43′22.51″E). Sediments were sampled at a water depth of 3.7 m. The detailed locations of the samples have been shown in Supplementary Fig. 1 in the Supporting Information.
Access & import/export	The collection of samples did not involve sensitive or restricted areas.
Disturbance	No disturbance caused by this study.

Reporting for specific materials, systems and methods

We require information from authors about some types of materials, experimental systems and methods used in many studies. Here, indicate whether each material, system or method listed is relevant to your study. If you are not sure if a list item applies to your research, read the appropriate section before selecting a response.

Materials & experimental systems

n/a	Involved in the study
☒	☐ Antibodies
☒	☐ Eukaryotic cell lines
☒	☐ Palaeontology and archaeology
☒	☐ Animals and other organisms
☒	☐ Clinical data
☒	☐ Dual use research of concern
☒	☐ Plants

Methods

n/a	Involved in the study
☒	☐ ChIP-seq
☒	☐ Flow cytometry
☒	☐ MRI-based neuroimaging

Plants

Seed stocks	N/A
Novel plant genotypes	N/A
Authentication	N/A
